# DI-5-Cuffs: Bone Remodelling and Associated Metabolism Markers in Humans After Five Days of Dry Immersion to Simulate Microgravity

**DOI:** 10.3389/fphys.2022.801448

**Published:** 2022-04-27

**Authors:** Marie-Thérèse Linossier, Laura Peurière, Peter Fernandez, Myriam Normand, Arnaud Beck, Marie-Pierre Bareille, Christine Bonneau, Guillemette Gauquelin-Koch, Laurence Vico

**Affiliations:** ^1^ INSERM, U 1059, University of Saint-Etienne, University of Lyon, Saint Etienne, France; ^2^ Institute of Space Physiology and Medicine (MEDES), Toulouse, France; ^3^ Biochemical Analysis Laboratory, University Hospital, Saint Etienne, France; ^4^ French Space Agency (CNES), Paris, France

**Keywords:** simulated microgravity, dry immersion, thigh cuffs, bone remodelling, energy metabolism

## Abstract

**Background:** The dry immersion (DI) model closely reproduces factors of spaceflight environment such as supportlessness, mechanical and axial unloading, physical inactivity, and induces early increased bone resorption activity and metabolic responses as well as fluid centralization. The main goal of this experiment was to assess the efficacity of venoconstrictive thigh cuffs, as countermeasure to limit cephalad fluidshift, on DI-induced deconditioning, in particular for body fluids and related ophthalmological disorders. Our specific goal was to deepen our knowledge on the DI effects on the musculoskeletal events and to test whether intermittent counteracting fluid transfer would affect DI-induced bone modifications.

**Methods:** Eighteen males divided into Control (DI) or Cuffs (DI-TC) group underwent an unloading condition for 5 days. DI-TC group wore thigh cuffs 8–10 h/day during DI period. Key markers of bone turnover, phospho-calcic metabolism and associated metabolic factors were measured.

**Results:** In the DI group, bone resorption increased as shown by higher level in Tartrate-resistant acid phosphatase isoform 5b at DI_24h_. C-terminal telopeptide levels were unchanged. Bone formation and mineralization were also affected at DI_24h_ with a decreased in collagen type I synthesis and an increased bone-specific alkaline phosphatase. In addition, osteocalcin and periostin levels decreased at DI_120h_. Calcemia increased up to a peak at DI_48h_, inducing a trend to decrease in parathyroid hormone levels at DI_120h_. Phosphatemia remained unchanged. Insulin-like growth factor 1 and visfatin were very sensitive to DI conditions as evidenced by higher levels by 120% vs. baseline for visfatin at DI_48h_. Lipocalin-2, a potential regulator of bone homeostasis, and irisin were unchanged. The changes in bone turnover markers were similar in the two groups. Only periostin and visfatin changes were, at least partially, prevented by thigh cuffs.

**Conclusion:** This study confirmed the rapid dissociation between bone formation and resorption under DI conditions. It revealed an adaptation peak at DI_48h_, then the maintenance of this new metabolic state during all DI. Notably, collagen synthesis and mineralisation markers evolved asynchronously. Thigh cuffs did not prevent significantly the DI-induced deleterious effects on bone cellular activities and/or energy metabolism.

## Introduction

Lack of Earth gravity is associated with body deconditioning as demonstrated by changes in various physiological systems such as cardiac, muscle and bone functions (Shelhamer, et al., 2020). The dry immersion (DI) model is a unique analogue of reduced gravity by providing mechanical and axial unloading, advanced physical inactivity, as well as a lack of a supporting structure for the body. Indeed, for subject freely suspended in water, support load is spread nearly uniformly around the entire bodysurface. The absence of specific zones that carry the weight (like the back and sides of the torso in bedrest) creates a support deprivation state akin to weightlessness. Our previous experiment had been carried out on 12 subjects placed in immersion conditions for 3 days. This study allowed us to show that the effects of DI on musculoskeletal system could be more severe and/or earlier than in support-loaded bed rest (Demangel et al., 2017; Linossier et al., 2017). Thus, serum marker of bone resorption activity as assessed by tartrate-resistant acid phosphatase isoform 5b and N-terminal crosslinked telopeptide of type I collagen levels increased as early as DI_24h_. At the same time, total procollagen type I N- and C-terminal propeptides and osteoprotegerin, representing bone formation markers, decreased. At the bone level, loss induced by prolonged microgravity exposure is mainly seen in the weight-bearing segments of the skeleton, i.e., the lower limbs, with few differences at the non weight-bearing radius site (Vico et al., 2017). This differential bone response could be related not only to greater unloading for weight-bearing sites, but also to redistribution of tissue fluids towards the thoraco-cephalad regions. To prevent shift in fluids towards the head, thigh cuffs are used to sequester blood and fluids in the lower limbs by compressing blood vessels in the proximal parts of the thighs. First tested in 1984 during a 232-days space flight, thigh cuffs have proved effective in alleviating the symptoms associated with cephalad fluid shift in the early hours and days in space (Arbeille et al., 1999; Fomina et al., 2004). Therefore, since about thirty years, thigh cuffs are used by cosmonauts in flight as a routine countermeasure limiting fluid movement and improving their comfort. Cardiovascular changes occur early and lead to a new stable hemodynamic balance after a few days (Buckey, 2006). Influences of thigh cuffs on the cardiovascular system and haemodynamic changes have already been well studied during Head-Down Bed Rest (HDBR) (Arbeille et al., 1999; Custaud et al., 2000; Millet, et al., 2000; Pavy-Le Traon et al., 2001; Yao et al., 2008). However, in these studies, their effects are not always consistent. Based on the first four studies cited, using a thigh pressure of 30 mm Hg during 10 h daily, the effects of thigh cuffs on plasma volume, heart rate and spontaneous baroreflex slope (SBRs) have been proven insufficient to prevent orthostatic intolerance induced by 7 days of HDBR. Yao et al. (2008) indicated that daily use of thigh cuffs at 40 mmg Hg (10 h/day) during 10 days of HDBR did not completely prevent the decrease in haemodynamics of the right middle cerebral artery, but, contrary to the other studies, was effective in preventing orthostatic intolerance. DI induces prompt body fluids centralization, mainly due to hydrostatic squeezing of superficial tissues and vessels, and is therefore particularly adapted for rapid evaluation of countermeasures against fluid transfer and its consequences. Such countermeasures become a priority in preparation of deep space missions, since cephalad fluid shift might contribute to spaceflight associated neuro-ocular syndrome. This integrative study was primarily designed to test the efficacy of venoconstrictive thigh cuffs, as countermeasure to limit cephalad fluidshift, on DI-induced deconditioning, in particular for body fluids and related ophthalmological disorders (for results cf. Robin et al., 2020; Kermorgant et al., 2021). However, several physiological systems interact, including the cardiovascular and musculoskeletal systems. By revealing that the skeleton exerts an endocrine regulation of sugar homeostasis, the Karsenty’s team expands the biological importance of this organ and the understanding of energy metabolism (Lee et al., 2007; Schwetz, Verena et al., 2012). The interaction between the different systems are not only affected by fluid movements and/or hemodynamic changes, but also by bone factors such as osteocalcin known for its endocrine role on the energy metabolism. Therefore, all modifications of fluid transfers (whether it is a redistribution under microgravity model and/or a limitation of this redistribution with the addition of Thigh cuff to DI) can potentially change the effects exerted by the bone on the metabolism. To date, no study has considered the impact of thigh cuffs on the bone remodelling and associated metabolism markers in humans either in real or simulated microgravity. In rats, it has been shown that femoral vein ligation by increasing intraosseous pressure, induces increased bone mass in the hindlimb of suspended animals (Bergula et al., 1999).

Therefore, this 5-day DI offers a unique opportunity to test thigh cuff effects on the musculoskeletal events. The first aim of this study was to see if the DI-induced changes persist over a 5 days dry immersion with the same intensity than the one seen after 3 days. The daily use of venoconstrictive thigh cuffs should allow us to see if the limitation of fluid transfer for 10 h/day can modulate the effects induced by DI on bone remodelling in humans.

## Material and Methods

### Subjects

Twenty healthy men were recruited. Two subjects withdrew from the study before the start of their experimental period for reasons unrelated to the protocol. A total of eighteen subjects were included in the study. A period of 4 days before immersion was applied in order to proceed to the basal data collection (BDC). Subjects were randomly allotted at BDC-2 to Dry Immersion (DI) or Dry Immersion with thigh Cuffs (DI-TC) group (9/9 split). All subjects were informed about the experimental procedures and gave their written consent. The experimental protocol conformed to the standards set by the Declaration of Helsinki and was approved by the local Ethic Committee (CPP Est III: October 2, 2018, n° ID RCB 2018-A01470-55) and French Health Authorities (ANSM: August 13, 2018). ClinicalTrials.gov Identifier: NCT03915457.

Baseline group characteristics are detailed in Table 1. There was no significant difference between groups at baseline.

**TABLE 1:** Baseline group characteristics at BDC-2.

	Age (y)	Height (cm)	Weight (kg)	BMI (kg/m^2^)	(ml/min/kg)
Dry Immersion (DI) *n* = 9	33.4 ± 7.0	176 ± 6	74.5 ± 7.2	24.2 ± 1.8	46.5 ± 8.1
Dry Immersion with Thigh cuff (DI-TC) *n* = 9	33.8 ± 4.0	180 ± 4	74.4 ± 9.2	22.8 ± 1.8	46.9 ± 5.8

Values are mean ± SD. Unpaired *t*-test did not reveal significant difference between groups.

### General Protocol, dry Immersion Organization, Thigh Cuffs Countermeasure

**FIGURE 1:**
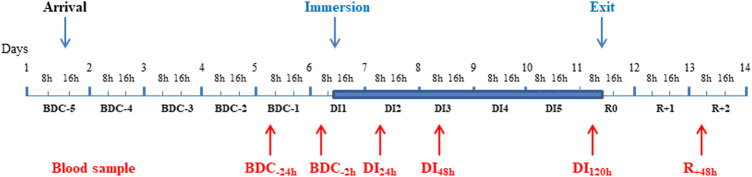
Description of the study. A Time line of the experiment with the timing of different blood samples. Blue area corresponds to the DI phase. BDC: baseline data collection; DI: dry immersion; R: recovery.

The study was conducted at the MEDES space clinic, Toulouse, France from November, 19, 2018 to March, 23, 2019 in a period lasting 12 days as described in Figure 1. Subjects arrived in the evening of BDC-5 and left after 48-h of recovery (at R + 2). The experimental protocol included 4 days of ambulatory baseline measurements before immersion (BDC-4 to BDC-1), 5 days (120 h) of dry immersion (DI1 to DI5) and 3 days of ambulatory recovery (R0, R + 1, R + 2).

A week prior to beginning of the protocol the subjects went to MEDES for pre-immersion muscle biopsy and resting metabolic rate measurement.

Subjects of the Cuff group wore the thigh cuffs during the 5 days of DI, from 10:00 to 18:00 at DI1 and from 08:00 to 18:00 at DI2-DI5. Thigh cuffs were elastic strips, adapted to each subject to have the same effects on lower-limb distensibility as at counterpressure of about 30 mmHg. Individual adjustment was determined for each subject with calf plethysmography, performed in the supine position at BDC-2. At DI1, thigh cuffs were put on immediately prior to the onset of immersion at 10 h. 30 mmHg was selected, as it matches to the initial thigh cuff pressure used by cosmonauts and the pressure tested and evaluated during a 7-day HDBR held at MEDES in 1997–1998 (Arbeille et al., 1999; Custaud et al., 2000; Millet, et al., 2000; Pavy-Le Traon et al., 2001).

A strict DI protocol was conducted according to methodology detailed previously (De Abreu et al., 2017; Linossier et al., 2017). Two subjects, one Dry Immersion and one Dry Immersion with Thigh Cuffs, underwent dry immersion simultaneously in the same room, in two separate baths (except for two subjects, one DI and one DI-TC, who had no mate). Thermoneutral water temperature was continuously maintained at 33 ± 0.5°C. Light-off period was set at 23:00–07:00. Daily hygiene, weighing and some specific measurements required extraction from the bath. During these out-of-bath periods, subjects maintained the -6° head-down position in order to avoid orthostatic stimulation and preserve DI effects as much as possible. Total out-of-bath supine time for the 120 h of immersion was 9.7 ± 1.3 h. On DI1-DI4 out-of-bath time was 1.1 ± 0.6 h/day. On DI5 out-of-bath time was 5.3 ± 1.1 h, because of muscle biopsy and MRI. Otherwise, during DI, subjects remained immersed in a supine position for all activities and were continuously observed by video monitoring. Body weight, blood pressure, heart rate and tympanic body temperature were measured daily. Water intake throughout the protocol was *ad libitum* between 35–60 ml/kg/day and was measured. The meals of each experiment day were identical for all participants and dietary intake was individually tailored and controlled during the study. VO_2max_ bicycle ergometer test was performed in the evening of BDC-2 and R0.

### Blood Sampling

Fasting blood samples were collected always at the same time (around 7.30 am) during the course of the study. As mentioned in Figure 1, they were taken at baseline 1 day (BDC_-24h_) and just before the commencement of immersion (BDC_-2h_), after 24 h- (DI_24h_), 48 h- (DI_48h_), and 120 h- (DI_120h_) immersion, then 48 h after the return to loading conditions (R_+48h_). Serum was separated after blood collection on BD Vacutainer^®^ SST™ tubes (with clotting activator). BD Vacutainer^®^ EDTA K3 tubes were used for plasma generation. Serum and plasma were frozen at −80°C right away after the centrifugation until analysis. Measurements were carried out simultaneously in all samples at the end of the study.

### Measurements of Biological Markers

All parameters were measured at BDC_-24h_ and BDC_-2h_ (2 h before immersion), then at DI_24h_, DI_48h_, DI_120h_ and R_+48h_ as indicated in Figure 1. It should be noted that only the data obtained at BDC_-24h_ were retained for the determination of the baseline level (BDC) ; indeed, the day before immersion, after the fasting blood sample taken at BDC_-24h_, a test to measure the metabolic flexibility by indirect calorimetry during the fasted to fed transition was made ; this test could potentially be at the origin of the systematically and significantly lower values at BDC_-2h_ vs. BDC_-24h_ recorded for the majority of markers.

Serum samples: C-terminal crosslinked telopeptide of type I collagen [CTx], procollagen type I N-terminal propeptide [P1NP], bone alkaline phosphatase [bAP], intact and N-mid osteocalcin fragment [OC] and insulin-like growth factor 1 [IGF1] were determined by automated chemiluminescence immunoassay (IDS-iSYS automated analyzer, Boldon, United Kingdom). The following parameters were measured by enzyme-immunoassay (EIA) kits: tartrate-resistant acid phosphatase isoform 5b [TRAP5b] (Microvue Bone, Quidel Corporation, San Diego, CA, United States ); undercarboxylated [Glu-OC] and carboxylated [Gla-OC] OC (Takara Bio, Inc., Otsu, Japan); secretory form of nicotinamide phosphoribosyl-transferase [visfatin] (Adipogen AG, Liestal, Switzerland); Irisin (Adipogen AG, Liestal, Switzerland); periostin (Biomedica Medizinprodukte GmbH, Wien, Austria). Serum intact parathyroid hormone [1–84 PTH], calcium and phosphorus were measured using electrochemiluminescence immunoassay (Cobas^®^8000 modular analyzer, Roche Diagnostics Ltd., Rotkreuz, Switzerland).

Plasma samples: Neutrophil gelatinase-associated lipocalin [lipocalin-2] was measured also by EIA kit (Epitope Diagnostics, Inc., San Diego, CA, United States.

### Statistical Analysis

In each group, for each parameter, baseline level (BDC) was defined as the value of the measured variables at BDC_-24h_. Because of non-normal distributions and small number of subjects, non-parametric statistics have been performed. Data were expressed as median and interquartile range (IQR).

In each group, the time effect was assessed using non-parametric Friedman rank-sum test. Wilcoxon tests corrected by the fdr adjustment method of Benjamini and Hochberg (1995) were used for post hoc comparisons between each timing.

The TC treatment effect was further analyzed at each time point by Mann-Whitney tests between the 2 groups.

The changes from baseline expressed as percent difference were plotted at DI_24h_ to DI_120h_ or R_+48h_ for all parameters in figures for better visualization.

The relationships between changes (expressed vs. BDC_-24h_) in parameters at DI_48h_ or at DI_120h_ were investigated using nonparametric Spearman correlations. All statistical tests were carried out with the R statistic software supported by the R Foundation for Statistical Computing. *p* values less than 0.05 were considered to be statistically significant.

## Results

### Body Weight and VO_2max_

Similar changes were recorded for the two groups. Body weight was found significantly decreased after 1-day immersion and remained so during all the immersion period (Table 2). Body weight did not fully normalize after 2 days reloading.VO_2max_ decreased during dry immersion phase by near 10% vs. BDC values.

**TABLE 2:** Body weight and VO_2max_ before and at the end of dry immersion.

Variable	Dry immersion (DI)				Dry immersion with thigh cuffs (DI-TC)			
	BDC	DI24h	DI120h	R+48h	BDC	DI24h	DI120h	R+48h
Morning weight, kg	73.5	72.6^a^	71.8^a,b^	72.8^a,b,d^	80.2	78.7^a^	78.3^a,b^	79.4^a,d^ (58.2–81.8)
	(63.5–87.7)	(62.6–86.4)	(62.6–86.4)	(62.5–86,0)	(59.3–82.9)	(58.5–82.0)	(58.0–81.1)	
VO_2max_, ml/mn/kg	47.3		42.7^a^’		46.9		43.1^a^	
	(33.7–57.8)		(31.8–53.4)		(36.6–55.1)		(37.2–49.0)	

Measurements were made at baseline (BDC), during dry immersion (DI) and after recovery. Data are expressed as median (interquartile range) for *n* = 9 per group. a, b, d: significantly different (*p*p < 0.05) vs. BDC, DI24 h, DI120 h, respectively. a: trend to significance to BDC There was no difference between DI and DI-TC groups.

### Bone Metabolism

For the different markers, data measured at baseline, during the immersion period and after 24 h of recovery are summarized in Tables 3 and 4. For all parameters, there was no significant difference between groups at baseline.

**TABLE 3:** Serum markers for bone cellular activities in Dry Immersion (DI) and Dry Immersion with Thigh Cuffs (DI-TC) during the 5-day dry immersion and after 48 h- recovery.

		BDC	DI_24h_	DI_48h_	DI_120h_	R_+48h_
**Markers for bone turnover**						
**Resorption activity**						
TRAP5b (U/L)	DI	2.79(0.80–4.33)	3.12(0,93–4.88)^a^	3.50(0.96–4.98)^a,b’^	3.11(1.08–5.12)^a^	3.44(1.05–4.94)^a^
	DI-TC	2.72(1.47–4.60)	2.93(1.80–6.60)	3.02(1.92–5.90)^a^	3.05(1.90–5.89)^a^	3.01(1.80–5.75)
CTx (pmol/L)	DI	6229(1655–9831)	6259(1390–11012)	5977(1619–10180)	5811(1840–11746)	5977(2005–10546)
	DI-TC	5638(2237–9460)	5966(2030–9436)	5830(1958–9780)	5480(1954–9505)	6613(2734–10839)
**Formation activity**						
P1NP (µg/L)	DI	67.2(37.7–109.6)	61.3(30.6–100.3)^a^	59.8(35.0–103.1)^a^	55.7(29.7–102.7)^a^	58.6(32.8–94.9)^a,b^
	DI-TC	92.5(52.3–136.1)	84.1(45.5–114.7)^a^	81.1(42.2–110.6)^a^	68.9(41.4–110.6)^a^	76.3(38.7–107.7)^a^
bAP (µg/L)	DI	16.0(10.8–20.7)	17.1(11.2–21.8)^a^	17.6(12.8–25.7)^a,b’^	16.5(10.4–22.1)^a,c^	14.8(10.4–22.1)^b,c,d^
	DI-TC	21.4(9.0–27.3)	23.1(9.8–27.4)^a’^	23.4(10.8–28.0)^a^	22.4(9.9–27.7)^b’,c^	20.2(8.6–25.3)^b,c,d^
Intact OC (ng/ml)	DI	23.7(17.9–39.3)	24.7(15.8–44.1)	24.2(17.6–44.7)	21.0(11.6–39.7)^a,b,c^	21.2(14.8–39.3)^b,c^
	DI-TC	27.3(13.6–39.5)	26.6(12.3–39.9)	29.3(12.4–35.4)	23.3(9.9–30.4)^a,b,c^	24.2(10.4–31.7)^a,b’,c^
Gla-OC (ng/ml)	DI	11.9(9.4–16.8)	13.3(9.4–18.0)	12.8(8.5–20.0)	11.8(6.5–18.8)^b,c^	11.2(7.9–17.4)^a,b,c^
	DI-TC	12.0(7.3–15.8)	11.8(7.6–14.6)	11.9(7.8–16.2)^b^	10.4(5.8–15.5)^c^	11.3(6.6–15.5)^c^
Glu-OC (ng/ml)	DI	7.5(2.9–17.5)	7.6(3.7–15.5)	8.2(3.4–17.5)	5.7(2.9–16.7)^c^	5.3(3.6–12.8)^b,c^
	DI-TC	7.6(4.3–16.3)	8.5(4.4–15.0)	9.3 (5.2–17.2)^b^	7.3(3.4–11.8)^b’,c^	6.9(3.5–13.1)^c^
**Osteocyte activity**						
Periostin (pmol/L)	DI	990(615–1137)	934(577–1074)	708(592–990)^a,b^	712(493–977)^a,b^	904(710–1138)^c,d^
	DI-TC	740(518–1147)	835(631–1122)	729(508–1163)	742(505–1061)	713(589–1174)

Measurements were made at baseline (BDC), during dry immersion (DI) and after recovery. Data are expressed as median (interquartile range) for *n* = 9 per group. a,b,c,d: significantly different (*p* < 0.05) vs. BDC, DI_24h_, DI_48h_, DI_120h_, respectively. a’,b’: trend to significance (*p* ≤ 0.07) vs. BDC, DI_24h_, respectively. There is no difference between DI and DI-TC groups. TRAP5b: tartrate-resistant acid phosphatase isoform 5b; CTx: C-terminal crosslinked telopeptide of type I collagen; P1NP: procollagen type I N-terminal propeptide; bAP: bone alkaline phosphatase; OC: osteocalcin; Gla-OCand Glu-OC: carboxylated and uncarboxylated osteocalcin, respectively.

**TABLE 4:** Serum markers for phospho-calcic status and metabolic regulators in Dry Immersion (DI) and Dry Immersion with Thigh Cuffs (DI-TC) during the 5-day dry immersion and after 48 h- recovery.

		BDC	DI_24 h_	DI_48 h_	DI_120 h_	R _+ 48 h_
**Markers for phospho-calcic metabolism**						
PTH (ng/L)	DI	23.4(17.2–46.5)	28.0(16.0–40.5)	27.4(14.7–47.1)	21.2(14.5–33.4)^a’,b’^	23.4(13.0–40.6)^b’^
	DI-TC	31.1(17.7–43.5)	31.2(19.0–44.2)	28.9(18.4–45.8)	27.5(20.2–35.7)	28.4(19.1–37.2)
Calcium (mg/L)	DI	96.0(94.0–102.4)	100.0(95.6–105.6)^a^	101.6(96.0–106.8)^a,b’^	99.6(96.0–105.2)^a^	96.4(90.8–100.4)^b,c,d^
	DI-TC	95.6(92.8–100.4)	96.8(94.4–102.8)^a,†^	98.0(94.0–106.0)^a,†^	96.4(92.4–100.8)^*^	92.8(90.8–98.8)^b,c,d^
Phosphorus (mg/L)	DI	38.1(32.6–49.9)	38.1(32.6–49.3)	37.8(33.8–48.1)	37.5(33.5–47.4)	36.9(32.9–47.4)
	DI-TC	38.8(28.2–43.4)	38.1(30.1–47.1)	39.4(31.3–44.3)	37.8(29.5–45.6)	38.1(31.3–44.6)
**Metabolism regulators**						
Visfatin (ng/ml)	DI^n^	0.85(0.22–1,52)	0.97(0.43–3.64)^a’^	1.20(0.72–6.93)	1.98(0.66–2.90)^a’^	1.21(0.13–2.62)^d’^
	DI-TC	0.79 (0.40–2.19)	1.73(0.42–3.32)^a’^	1.06(0.43–4.58)^a’^	2.11(0.37–3.79)^a^	0.77(0.30–2.24)^b,c,d^
Lipocalin-2 (ng/ml)	DI	148(102–235)	148(102–224)	163(119–205)	161(115–237)	161(113–242)
	DI-TC	173(113–191)	152(125–192)	159(126–193)	162(124–201)	164(120–194)
IGF1 (µg/L)	DI	223(168–301)	225(189–292)	239(208–323)^a,b^	246(181–318)^a^	194(144–275)^a,b,c,d^
	DI-TC	220(141–242)	232(128–245)^a’^	231(132–252)^a’^	208(114–250)^b’,c, *^	172(114–205)^a,b,c,d^
Irisin (µg/ml)	DI	6.0(2.7–15.2)	7.0(4.1–12.7)	6.0(2.9–15.7)	6.2(3.2–13.4)	5.7(2.6–13.5)
	DI-TC	5.0(3.0–9.4)	5.3(3.7–10.2)	5.3(3.4–9.8)	4.1(2.9–8.4)	4.3(2.6–11.3)

Measurements were made at baseline (BDC), during dry immersion (DI) and after recovery. Data are expressed as median (interquartile range) for *n* = 9 per group, except for Visfatin (^n^: analysis made for n = 8 only for DI group). a,b,c,d: significantly different (*p* < 0.05) vs. BDC, DI_24h_, DI_48h_, and DI_120h_, respectively ; a’,b’,c’,d’: trend to significance (*p* < 0.07) vs. BDC, DI_24h_, DI_48h_, and DI_120h_, respectively ; † and *: trend (*p* < 0.10) and significant difference (*p* < 0.05) vs. DI group, respectively. PTH: parathyroid hormone; IGF1: insulin growth factor 1.

**FIGURE 2:**
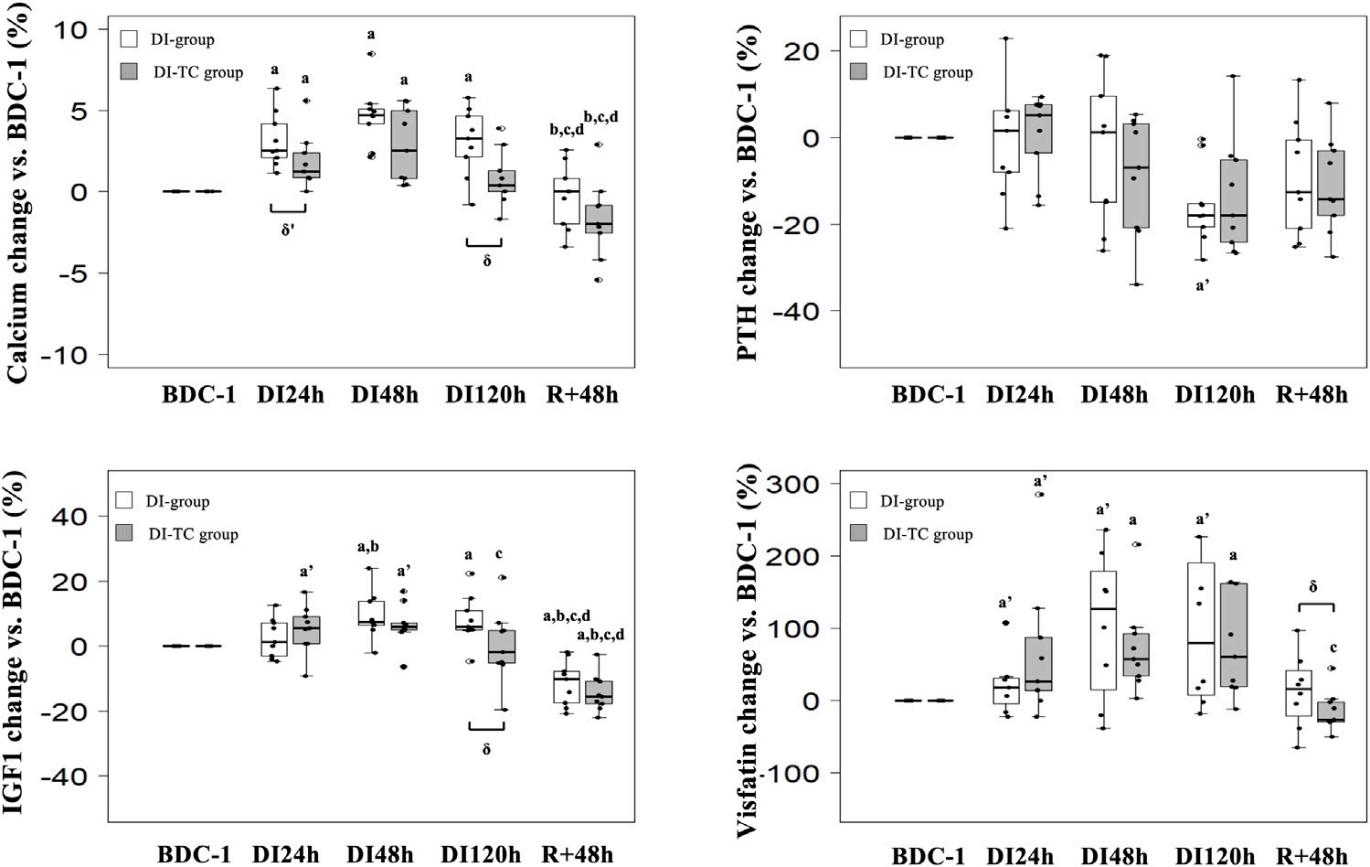
Effect of Thigh cuff on dry immersion-induced changes on the metabolic response. *At Top*, changes in calcic metabolism. *At bottom*, changes in values of IGF1 and visfatin. Values are medians ± interquartile range for *n* = 9, except for Visfatin (analysis made for *n* = 8 only for DI group). a,b,c,d indicate significant differences vs. BDC, DI_24h_, DI_48h_ and DI_120h_, respectively. a’: trend to significance (*p* < 0.10) vs. BDC. δ indicate significant differences (*p* ≤ 0.05) between DI and DI-TC groups.

**Phosphocalcic metabolism**: Calcium significantly increased as soon as the first day of immersion and reached a peak at DI_48h_ in the two groups; at DI_48h_, these levels were higher by 5 and 3% for DI and DI-TC, respectively, when compared to BDC, this increase being less in the DI-TC than DI (Figure 2). These levels remained elevated during all dry immersion phase for DI group whereas, for DI-TC group, concentrations returned to BDC level at DI_120h_; at this latter timing, calcium concentrations became significantly higher for DI vs. DI-TC.

Serum intact PTH levels tend to decrease at DI_120h_ by 18% in DI group (*p* = 0.064) when compared to BDC while no significant change was observed in DI-TC group. Phosphatemia was unchanged during the immersion phase in the two groups.

**FIGURE 3:**
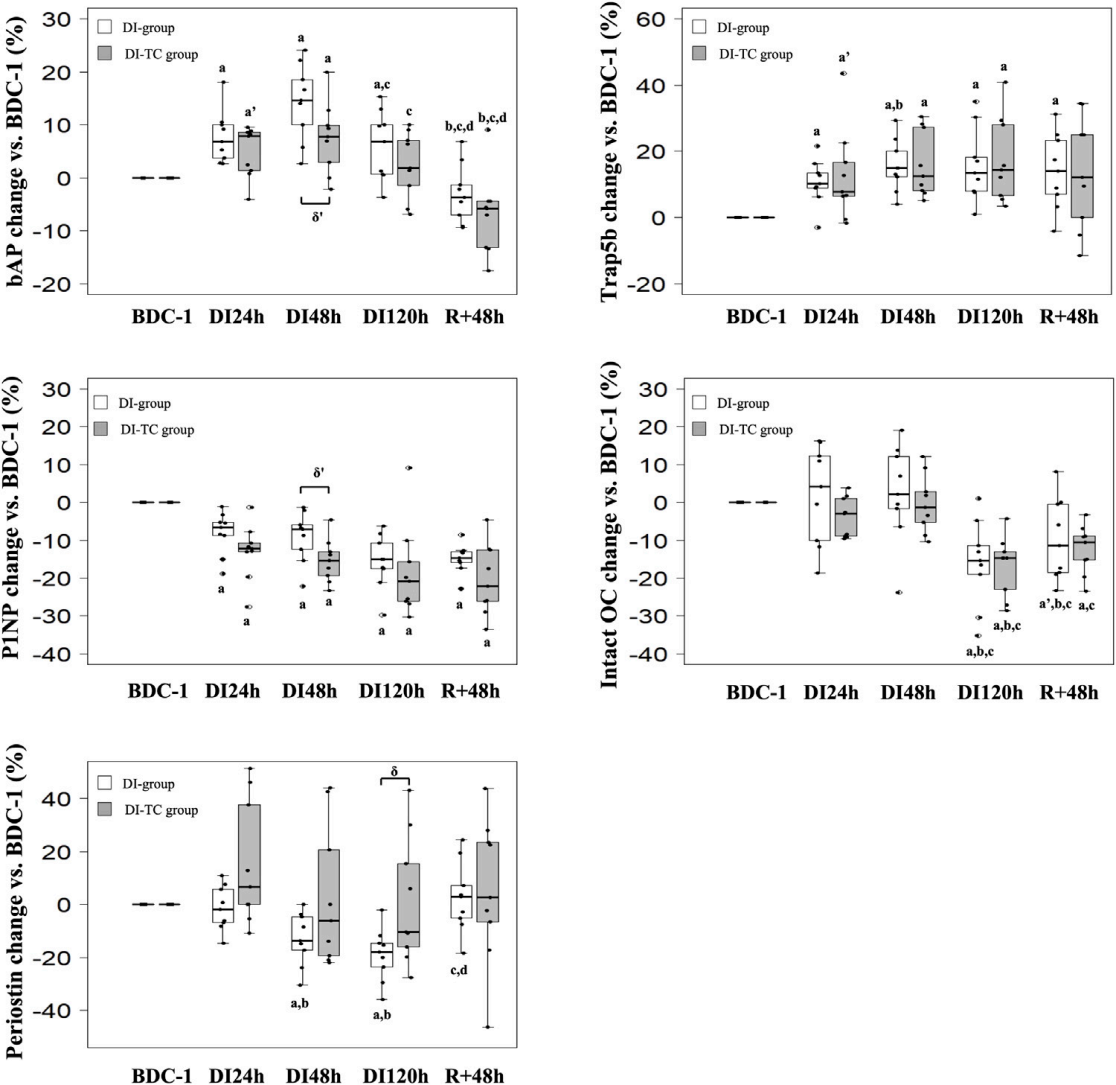
Effect of Thigh cuff on dry immersion-induced changes on bone metabolism. Changes in bone resorption and formation or mineralisation markers during dry immersion without (white) or with (grey) thigh cuff. Values are expressed as medians ± interquartile range for *n* = 9 per group. a,b,c,d indicate significant differences vs. BDC, DI_24h_, DI_48h_ and DI_120h_, respectively. a’ indicate trend to significance vs. BDC. δ‘ and δ indicate trend (*p* < 0.10) or significant differences (*p* ≤ 0.05) between DI and DI-TC groups. TRAP5b: tartrate-resistant acid phosphatase isoform 5b; P1NP: procollagen type I N-terminal propeptide; bAP: bone alkaline phosphatase; intact OC: intact osteocalcin.

**Bone resorption activity**: Serum TRAP5b significantly increased as soon as the first day of immersion and remained elevated during the entire dry immersion phase (by 15 and 13% at DI_48h_ for DI and DI-TC, respectively when compared to BDC) in both groups (Figure 3). After the DI period, at R_+48h,_ TRAP5b remained high in the DI-group while in the DI-TC it began to decrease, the difference between the two groups being not statistically different. No change in serum CTx was seen in the two groups (Table 3).

**Bone formation activity**: P1NP decreased progressively during all immersion periods in the two groups; at DI_48h_, the decrease appeared slightly smaller for DI vs. DI-TC although not significantly (*p* = 0.063); at DI_120h_, levels were significantly lower by 15 and 21% for DI and DI-TC, respectively when compared to BDC (Figure 3). Serum bAP concentrations increased as early as 1-day immersion and reached a peak at DI_48h_ ; this increase appeared slightly higher for DI when compared to DI-TC although not significantly (15 vs. 8%, respectively, *p* = 0.063) ; levels remained elevated during all dry immersion phase for DI group wheras, for DI-TC group, concentrations returned to BDC level at DI_120h_ ; after 48-h of recovery, bAP rates were 4 and 6% lower vs. BDC in DI and DI-TC, respectively (Figure 3).

Similarly, in the two groups, total intact OC level was maintained during the first 48 h of dry immersion; then, a decrease was recorded such as, at DI_120h_, the concentrations were 15% lower than BDC values. 48 h of recovery are insufficient to recover the baseline levels tending to keep concentrations at R_+48h_ 10% lower than BDC values for both DI and DI-TC. Despite the lack of statistical significance, Gla-OC evolved in a similar way to total intact OC for both groups. No significant change was observed in its undercarboxylated forms (Glu-OC) or in the Glu/Gla-OC ratio during all the study.

**Osteocyte activity**: Periostin, a gla domain vitamin K dependent factor, is involved in many processes among which the remodelling of bone tissue as a response to injury. In DI group, circulating periostin concentrations progressively decreased during all immersion period reaching, at DI_120h_, levels equal to 82% of BDC rates; then these values returned to baseline level after 48 h of recovery (Figure 3). When thigh cuff was associated with dry immersion, a similar decrease than in DI was observed, although not reaching statistical difference; nevertheless, the decrease in periostin at DI_120h_ was significantly different in DI vs. DI-TC (*p* = 0.050).

### Energy Metabolism and Hormones Levels

Visfatin concentrations increased as early as after 24 h of dry immersion for the majority of subjects regardless of treatment. It continued to increase throughout the dry immersion phase until reaching, at DI_48h_, higher levels by 120 and 60% for DI and DI-TC respectively, when compared to BDC (Figure 2); after 48 h of recovery, the level remained above the BDC values for 7 of DI subjects whereas the concentrations returned to or even decreased below the basal values for DI-TC group. Furthermore, circulating IGF1 levels progressively increased during immersion in both groups until reaching, at DI_48h_, 8 and 6% higher concentrations for DI and DI-TC, respectively, when compared to BDC ; levels remained elevated during all dry immersion phase for DI group wheras, for DI-TC group, concentrations returned to BDC level at DI_120h_ ; at this latter timing, IGF1 concentrations became significantly higher for DI vs. DI-TC ; after 48-h of recovery, circulating IGF1 levels were lower vs. BDC by 10 and 16% in DI and DI-TC, respectively.

Lipocalin-2 and irisin, two potential regulators of bone homeostasis, were unchanged for both groups during the entire experiment.

### Associations Between Biochemical Blood Markers

**At baseline**, significant correlations were found between bone resorption markers (r = 0.785, *p* = 0.0001 between CTx and Trap5b) and bone formation markers (r = 0.811, *p* < 0.0001 between intact OC and P1NP). In addition, collagen bone formation and mineralisation processes were also coupled (r = 0.683, *p* = 0.002 between P1NP and bAP). Moreover, statistical analysis elucidated the coupling between resorption and formation as shown by the relationships between intact OC vs. CTx and Trap5b (r = 0.793, *p* < 0.0001 and r = 0.669, *p* = 0.002, respectively) or between P1NP vs. CTx and Trap5b (r = 0.606, *p* = 0.008 and r = 0.650, *p* = 0.004, respectively). Furthermore, Glu-OC concentrations were linked to those of CTx and intact OC levels (r = 0.577, *p* = 0.012 and r = 0.666, *p* = 0.0026, respectively).

**Changes in parameters at DI_48h_ and/or DI_120h_**: Since thigh cuff had practically no significant effects on the measured parameters, relationships between their changes were established from all subjects (with or without thigh cuff i.e., 17 or 18 subjects) due to the dry immersion effect; they were summarized in Table 5.

**TABLE 5:** Association between biochemical blood markers.

Markers		Trap5b	CTx	Glu-OC	bAP	P1NP	Int OC	Gla-OC	Periostin	IGF1	Visfatin	Calcium	PTH	Phosphorus	Irisin
Trap5b	BDC value		0.79	-	-	0.65	0.67	-	-	-	-	-	-	-	-
	Δ at DI48h		-	-	-	-	-	-	-	-	−0.50	-	-	-	-
	Δ at DI120h		-	0.61 **	0.62	-	-	-	-	-	-	-	-	-	-
CTx	BDC value			0.58 *	-	0.61**	0.79	-	-	-	-	-	-	-	-
	Δ at DI48h			0.58 *	-	-	-	-	-	-	-	-	-	-	-
	Δ at DI120h			0.59 **	-	-	-	-	-	-	-	-	-	0.48 *	0.54 *
Glu-OC	BDC value				-	-	0.67**	-	-	-	-	-	-	-	-
	Δ at DI48h				-	-	0.48*	-	-	0.52 *	-	-	-	-	-
	Δ at DI120h				-	-	0.52 *	0.53 *	-	-	-	-	-	-	-
bAP	BDC value					0.68	-	-	-	-	-	-	-	-	-
	Δ at DI48h					-	-	-	-	0.56 *	-	-	-	-	-
	Δ at DI120h					-	-	-	−0.60 **	0.51 *	-	-	-	-	-
P1NP	BDC value						0.81	-	-	-	-	-	-	-	-
	Δ at DI48h						0.52*	-	-	-	-	0.58 *	-	−0.50 *	-
	Δ at DI120h						0.51 *	0.65**	-	-	-	-	-	-	-
int OC	BDC value							-	-	-	-	-	-	-	-
	Δ at DI48h							-	-	-	-	-	-	-	-
	Δ at DI120h							0.71 **	-	-	-	-	-	-	-
Gla-OC	BDC value								-	-	-	-	-	-	-
	Δ at DI48h								-	-	-	-	-	-	-
	Δ at DI120h								-	-	-	-	−0.48	-	-
Periostin	BDC value									-	-	-	-	-	-
	Δ at DI48h									-	-	-	-	-	-
	Δ at DI120h									-	-	-	-	-	0.54 *
IGF1	BDC value										-	-	-	-	-
	Δ at DI48h										-	-	-	-	-
	Δ at DI120h										-	0.61 **	-	-	-
Visfatin	BDC value											-	-	-	-
	Δ at DI48h											0.52 *	-	−0.52 *	-
	Δ at DI120h											-	-	-	-
Calcium	BDC value												-	-	-
	Δ at DI48h												−0.60	-	-
	Δ at DI120h												-	-	-
PTH	BDC value													-	-
	Δ at DI48h													-	-
	Δ at DI120h													-	-
Phosphorus	BDC value														-
	Δ at DI48h														-
	Δ at DI120h														0.52 *
Irisine	BDC value														
	Δ at DI48h														
	Δ at DI120h														

Spearman correlation coefficients between values at BDC or between percent changes at DI_48h_ or DI_120h_ in bone and energy metabolism parameters for *n* = 18 except for Visfatin which was made for *n* = 17. Changes are expressed in percent from BDC. The values of Spearman r were indexed by the significance (*, ** or *** for p value < 0.05, 0.01 or 0.001, respectively).

Whatever the duration of dry immersion, no correlation was found between changes in CTx and Trap5b.

In the early dry immersion phase (at DI48 h exclusively), the following relationships were obtained: 1) ΔGlu-OC positively with ΔIGF1; 2) ΔVisfatin positively with ΔCalcium but negatively with ΔTrap5b and ΔPhosphorus; 3) ΔP1NP positively with ΔCalcium but negatively with ΔPhosphorus; 4) ΔCalcium negatively with ΔPTH.

In the later dry immersion phase (at DI120 h), ΔGla-OC has been correlated positively with ΔP1NP, ΔintactOC and ΔGlu-OC but negatively with ΔPTH; In the same time, ΔCTx with ΔPhosphorus, ΔIrisine with both ΔCTx, ΔPhosphorus and ΔPeriostin; ΔbAP positively with ΔTrap5b but negatively with ΔPeriostin; ΔCalcium with ΔIGF1; ΔGlu-OC with ΔTrap5b.

After both 48 and 120 h of dry immersion, changes in P1NP and intact OC were positively correlated. It is also worth noting the positive relationships between: 1) ΔGlu-OC (decarboxylated OC form) and both Δintact OC and ΔCTx; 2) ΔbAP and ΔIGF1.

## Discussion

Our current results confirmed that the microgravity simulated by short-term DI model challenges bone remodelling activity in human (Linossier et al., 2017). It demonstrated rapid bone adaptation with a metabolic peak at 48 h of DI followed by the maintenance of a dissociation between bone formation and resorption beyond the end of the dry immersion conditions. Despite beneficial effects induced by thigh cuff on body fluid changes (Robin et al., 2020), this countermeasure did not prevent the deleterious effects in bone cellular activities.

### Dry Immersion Conditions and Bone Remodelling Activity

As early as 24-h DI, resorption evaluated from circulating Trap5b level was already elevated by 7%. After 48 h of immersion, this marker continues to increase up to 14%, which is greater than the 7% observed at the same time in the first experiment of 3 days (Linossier et al., 2017). Regarding bone formation activity, P1NP concentrations indicative of lower type 1 collagen deposition decreased while bAP, an enzyme promoting bone mineralisation, increased, as also observed at DI_48h_ in the shorter 3-d study. Our current results confirmed that the microgravity simulated model by short-term DI challenges bone remodelling activity.

Sclerostin, an osteocyte-secreted protein negatively regulating osteoblast activity and therefore bone formation, has been shown to increase from 8 to 10 days of bedrest (Frings-Meuthen et al., 2013) suggesting that it is a late responding marker. In line with these results, we did not detect any change in the 3-day dry immersion experiment (Linossier et al., 2017) and did not analyzed it in the present study. However, we assessed periostin, a matricellular protein of cortical bone and periosteum and found that its serum level decreased as early as 48 h in DI group. During hindlimb suspension in mice periostin expression decreased and contributed to cortical bone loss through an increase in sclerostin (Gerbaix et al., 2015). Periostin-deficient mice have low bone mass and respond less to physical activity due to a lack of sclerostin inhibition by mechanical loading (Bonnet et al., 2009, 2012). After 4.5–6-months spaceflight in cosmonauts, periostin serum level was showed to predict tibia cortical evolution during space mission (Vico et al., 2017). In the current 5-d DI, periostin drop may thus be indicative of a later reduction in its antisclerostin activity.

Extending the dry immersion from 3 to 5-days revealed adaptation kinetics with bone marker changes for Trap5b, bAP, P1NP and periostin reaching a peak at 48 h of DI followed by stabilization at this new steady-state throughout unloading condition. The DI-induced alteration in bone formation activity was confirmed by the significant drop in total OC and its carboxylated form (Gla-OC) at DI_120h_. Changes in bone formation markers (P1NP, OC, and Gla-OC) were correlated with each other. The evolution of total OC seems to result also from the change in its decarboxylated forms: when considering the 18 subjects together, whereas Glu-OC levels were found higher at DI_48h_, they decreased significantly after 5 days of DI, as did total OC.

This dissociation between bone cellular activities supports the rapid onset of bone alterations. Serum markers give a global trend that might result from local changes as evidenced by some authors who reported, as early as 7 days of DI, a decrease in bone mineral density of the lower part of the skeleton (proximal epiphysis of the femur) to the benefit of the upper part (skull, hand, costal bones) when compared to baseline (Kotov et al., 2003).

### Dissociation of Serum Markers Within the Activities of Either Bone Formation or Bone Resorption

Regarding the bone formation markers and as in the previous 3-day campain, we observed a dissociation between the evolution of bone matrix synthesis and mineralisation markers. Indeed, serum P1NP and OC decreased whereas serum levels of bAP increased in the two groups. While, at baseline, these processes were quite well coupled as evidenced by the correlation established between P1NP and bAP, DI conditions would seem to have induced a decoupling. It would be interesting to see if such an adaptation exists in other unloading models.

Regarding the bone resorption markers and, contrary to the previous study, no change was observed in circulating CTx levels, even after 5 days of DI. Several explanations could be at the origin of the dissociation between changes in Trap5b and CTx. First, contrary to CTx, the Trap5b level is independent of renal function as it is rapidly inactivated during circulation by the loss of iron (Halleen et al., 2000). Renal function is altered by DI as evidenced by increased renal excretion of liquids and negative water balance (Larina and Kysto, 2008; Navasiolava et al., 2011; Noskov et al., 2011). This was confirmed in our subjects whose results were published previously by Robin et al. (2020). Therefore, in such situations, serum TRAP5b appeared to be a more relevant indicator than telopeptides of type I collagen for the estimation of bone resorption, as shown in hemodialysis patients (Shidara et al., 2008). Second, this dissociation between TRAP5b and CTx could be a timing issue. TRAP5b levels reflect the osteoclast numbers while CTx is released into the circulation in response to osteoclast function (Henriksen et al., 2007). At the basal level, our subjects were characterized by a 24% lower Trap5b activity when compared to the 1st experiment (*p* < 0.05) leading to suppose a lower number of osteoclasts whereas CTx levels were similar in the two studies. Therefore, it is possible that higher TRAP5b increases induced by DI conditions, bringing circulating levels at 48-h DI similar in the 2 studies, preceded those of CTx. For continuous bone remodelling and tissue reparation of the skeleton, the number of osteoclasts rather than their functional activity appeared essential to mediate the coupling between bone resorption and bone formation (Schaller et al., 2004; Karsdal et al., 2005; Koh et al., 2005; Martin and Sims, 2005). When we considered changes at DI_120h_ for all 18 subjects together, we found some relationships that seemed to agree with these latter ones. Indeed, changes in Trap5b are positively correlated with those of the mineralisation marker (bAP) and the decarboxylated OC form (Glu-OC). Despite the lack of significant variation in both CTx and Glu-OC in DI group, these 2 markers remain correlated whether on baseline or in terms of variations at both DI_48h_ and DI_120h,_ supporting a role for bone resorption in the decarboxylation of osteocalcin.

### Relationship Between Bone Remodelling and Associated Metabolic Responses

In our previous study, the increased resorption had been associated with the onset of insulin resistance along with a higher degree of OC decarboxylation and a higher circulating IGF1 (Linossier et al., 2017). In the present study, blood glucose was unmodified (Robin et al., 2020), but data on blood insulin are lacking. On the other hand, circulating IGF1 levels were increased by 8% for the DI group at DI_48h_, which could be the direct consequence of the stimulated GH secretion following a potential increase of the insulin response. When compared to the first dry immersion experiment, this activation was shifted by 24-h (IGF1 levels not yet increased at DI_24h_) and was less than the 15% higher IGF1 concentration observed at DI_48h_ in our previous study. Moreover, this could not be associated with a significant increase in degree of OC decarboxylation although 8 out of 9 subjects increased their Glu-OC concentration at DI_48h_ in DI group. Despite the small sample size, we showed that the higher the rates of IGF1, the higher the circulating rates of Glu-OC (r = 0.70, *p* = 0.0358 at DI_48h_ for DI group). One of the rare studies which have measured Glu-OC under unloading models (Morgan et al., 1985) reported no change while bone resorption activity was increased by 80% after 30 days of HDBR.

IGF1is known to stimulate bone formation. However, as already shown in Linossier et al. (2017), increased circulating IGF1 levels were associated with decreased bone formation markers under dry immersion. The present study is in line with Bikle and colleagues as well as Long and colleagues (Bikle et al., 1994, 2015; Long et al., 2011) showing that unloading would induce the development of resistance to this growth factor. Indeed, in rats submitted to hindlimb unloading (Bikle et al., 1994) observed the failure of the unloaded bone to grow in response to exogenous IGF1. In the same model, Long et al. (2011) showed that bone marrow osteoprogenitor cells originating from unloaded rats failed to respond to IGF-1 treatment *in vitro*.

### Comparison Between the Current 5-d dry Immersion and a Previous 3-d dry Immersion Might Explain Some Differences in Serum Marker Changes

As demonstrated for the first time during the previous 3-day dry immersion experiment, our results confirmed the great responsiveness for visfatin. However, as for IGF1, the visfatin increase was delayed by 24 h (Linossier et al., 2017). This shift in the timing of the metabolic response might be related to the difference in training level of the subjects between these two studies. We compared the characteristics of our subjects with those of the group from the first experiment published in 2017. It was found that, while no difference in baseline group characteristics related to age, height, weight and BMI were reported between these two studies, VO_2max_ was found to be 20% higher in the present study as compared to the previous one (*p* < 0.05). This better aerobic capacity was accompanied by visfatin rates 4 to 5 times lower at BDC_-1_ (*p* < 0.0001) when compared to the first experiment. In bed rest studies that consider alterations in bone cellular activities, no VO_2max_ data were reported. The value, reported elsewhere in HDBR studies (Capelli et al., 2006; Bringard et al., 2010), are comparable to those of the subjects of our first experiment. The VO_2max_ level of the subjects in this 2^nd^ experiment remains higher than in all these studies. Rare are studies that explored relationship between physical ability and visfatin levels in healthy men. Choi et al. (2007) reported that aerobic exercise training induced a decrease in plasma visfatin levels in Korean women. The lack of a consensus on the normal visfatin concentration and its relationship to anthropometric and metabolic parameters in adult has led Jurdana et al. (2013)) to study the association between increased circulating visfatin levels and anthropometric parameters in obesity. These authors evidenced that physical fitness was the best significant predictor of baseline visfatin concentration in male participants. Such observations could explain that, at BDC_-1_, visfatin levels were much lower for DI and DI-TC groups when compared with our previous study. Furthermore, when compared to the 1st experiment, the 18 subjects were characterized not only by a 24% lower Trap5b activity (*p* < 0.05) but also by a 22% higher osteocalcin level (*p* < 0.05) at BDC_-1_; this evidenced a different bone remodelling rate with lower bone resorption and higher bone formation activities in the present study when compared to 3-day DI experiment. Such differences are apparently due to better physical ability. In line with this statement (Gabel et al., 2021) recently reported in astronauts that pre-flight markers of bone turnover and exercise history may identify crewmembers at greatest risk of bone loss due to unloading.

### Reversibility of Dry Immersion-Induced Changes

Similar to bone cellular activities, associated metabolic processes responded with an early phase of adaptation with a peak at 48 h of DI that maintains at the new steady-state throughout unloading condition. Adaptation processes to unloading are partially reversible since, after 48 h of recovery, only some of the markers (i.e., Ca, PTH, bAP, periostin and visfatin) returned to their basal level. Switch from unloaded condition to 1g is associated with a persistent impairment of bone turnover markers (Trap5b, P1NP and OC and its forms) as well as a significant decline in circulating IGF1 concentrations below the basal level. The endocrine role of bone could again be responsible for the production of IGF1 below BDC as a result of reduced stimulation of Langerhans cells due to lower circulating Glu-OC levels.

### Do Thigh Cuffs Prevent the Early dry Immersion Induced Effects on Bone Remodelling Activity and Energy Metabolism: dry Immersion-TC vs. dry Immersion Group?

The effects of venoconstrictive thigh cuffs on body fluid changes from the subjects of this experiment were recently reported by Robin et al. (2020). These authors evidenced that the use of thigh cuffs was associated with a slowdown and limitation of the total body water loss and a trend to limit the decrease in plasma volume. Nevertheless, these beneficial effects of thigh cuff on body fluid changes remained very moderate and did not counteract decreased tolerance to orthostatic challenge. No study has considered the impact of thigh cuff on the bone cellular activities and muscle deconditioning induced by real or simulated microgravity. Here, thigh cuff is ineffective since metabolic profiles of bone resorption and formation remained similar between the two groups. At DI_48h_, the trends towards a lesser increase in bAP and a lower decrease in P1NP as well as a lower release of calcium under thigh cuff were probably more related to the limitation of plasma volume loss by 1/4–1/3 under thigh cuffs when compared to immersion alone (Robin et al., 2020). On the other hand, it is worth noting the absence of a significant decrease in periostin contrary to DI condition only under thigh cuffs. The application of thigh cuffs might have been more effective on energy metabolism in partially preventing the IGF1 stimulation and visfatin production. However, as for previous markers, differences in IGF1 being minimal between the two groups should be considered with great care. On the other hand, the difference in plasma volume cannot by itself explain the lower (but not significant) percentage increase in visfatin levels under thigh cuff vs. DI. (60 vs. 120%).

### Limitations

The main limitation of this study is the low number of subjects in each group (*n* = 9). The number of subjects was not specifically powered for bone metabolism criteria, but for change in optic nerve sheath diameter, a primary outcome measure for this protocol (ck Kermorgant et al., 2021). This might have been responsible for the lack of significant changes, as seen for example in Glu-OC since statistical difference was achieved with the unique group of 12 subjects in the 1st experiment (Linossier et al., 2017). Also, due to the numerous teams involved in this experiment, limited serum was available which prevented us from testing more parameters (such as sclerostin, markers of insulinic response or renal function).

## Conclusion

The present study confirmed the early adaptation of bone activities and increased visfatin levels under dry immersion conditions. It also allowed the possibility to substantiate the dissociation between bone resorption and formation, which was maintained throughout the immersion period with a metabolic peak at 48 hours and was not restored 48 h after the reloading. The disconnection observed between bone collagen synthesis and its mineralisation highlighted dysfunction in the bone formation phase. In view of the changes in fluid-volume homeostasis, it seemed relevant to question the impact of fluid transfers on the bone metabolism. However, thigh cuffs applied intermittently to reproduce in-flight use and avoid deleterious venous effects did not allow to prevent the early dry immersion induced effects on bone remodelling activity and energy metabolism. This suggests that body fluids modifications are not the leading events for bone changes under DI. Another DI effects, not counteracted by thigh cuffs, such as supportlessness, mechanical and axial unloading, and physical inactivity, seemingly dominate for bone and metabolic responses. This trial is the second DI study done in Europe and we believe that the data obtained are valuable to set up effective countermeasures for the effects of gravitational unloading.

## Data Availability

The original contributions presented in the study are included in the article/Supplementary Material, further inquiries can be directed to the corresponding author.
